# The Prevention of Disease in Armies in the Field

**Published:** 1904-09

**Authors:** 


**The Prevention of Disease in Armies in the Field.** By ROBERT CALDWELL, F.R.C.S., D.P.H., Major R.A.M.C. Pp. viii., 182. London: Baillière, Tindall & Cox. 1904.

This work will be found of interest and service not only to officers in the Royal Army Medical Corps and volunteer medical staff, for whom it is especially designed, but also to medical men generally, and all such as are interested in the physical well-being of the soldier on service. The great value of Major Caldwell's book lies in the fact that it is essentially modern and practical, founded on personal experience obtained in two of our largest campaigns, namely those in Egypt and South Africa. Enteric fever, the most destructive disease of camp life, naturally claims the greatest amount of attention, and the writer claims that the influence of water supply is of less importance than that of soil pollution in the causation of this disease. Indeed, the keynote of the book is the necessity for a cleanly soil for a healthy camp. Theoretically, there is much to support the view that bacteria of the colon type may undergo modification and excite such diseases as diarrhœa, continued fever, and enteric fever, and Major Caldwell's practical experience in South Africa strongly supports this view. The difficult problem of the disposal of camp refuse, so essential to the preservation of soil cleanliness, is exhaustively dealt with, and a diagram and description of the author's improvised refuse destructor should prove of service to any who are superintending military camps at home or abroad. In questions of military hygiene, such as clothing, marching, cooking, disinfection, &c., this will be found a handy work of reference, the ideas promulgated being scientifically sound and not slavishly moulded in the traditions of the service. The chapter on the sanitary organisation of a field force is likely to meet with sharp criticism from army medical officers. The numerous illustrations are interesting mementoes of a medical officer's work in the late South African campaign. We trust that this book will be extensively read, for the lamentable history of recent campaigns has shown that our army medical authorities have not yet appreciated the fact that in war, even more than in peace, the prevention of disease is more important than the cure.

The recent award of the Parkes prize will give this book the position it so well deserves.

